# Acute Medication Use in Patients With Migraine Treated With Monoclonal Antibodies Acting on the CGRP Pathway: Results From a Multicenter Study and Proposal of a New Index

**DOI:** 10.3389/fneur.2022.846717

**Published:** 2022-02-28

**Authors:** Lucia Sette, Valeria Caponnetto, Raffaele Ornello, Tomáš Nežádal, Dana Čtrnáctá, Jitka Šípková, Zuzana Matoušová, Simona Sacco

**Affiliations:** ^1^Neuroscience Section, Department of Applied Clinical Sciences and Biotechnology, University of L'Aquila, L'Aquila, Italy; ^2^Department of Neurology, 1st Faculty of Medicine, Military University Hospital Prague, Charles University, Prague, Czechia; ^3^Department of Neurology, 2nd Faculty of Medicine, Motol University Hospital Prague, Charles University, Prague, Czechia

**Keywords:** migraine acute treatment, medication, monoclonal antibodies, real-life, calcitonin gene-related peptide (CGRP)

## Abstract

**Introduction:**

Assessing the impact of migraine preventive treatments on acute medication consumption is important in clinical evaluation. The number of acute medication intakes per each monthly migraine day (MMD) could provide insights on migraine burden and represent a new proxy of treatment effectiveness in clinical trials and real-life studies. We evaluated the effect of monoclonal antibodies acting on calcitonin gene-related peptide (CGRP) pathway on the consumption of migraine acute medication in real-life.

**Methods:**

In two headache centers in Prague (CZ), we included and followed up to 6 months consecutive patients treated with MoAbs acting on CGRP (erenumab or fremanezumab). For each month of treatment, we reported monthly drug intake (MDI) in doses of any medication, migraine-specific (MS), and non-migraine-specific (non-MS) medications, and computed a ratio between MMDs and MDI, i.e., Migraine Medication Index (MMI) for MS and non-MS medications.

**Results:**

We included 90 patients (91.1% women) with a median age of 47 [interquartile range (IQR) 42–51] years; 81 (90.0%) treated with erenumab and 9 (10.0%) with fremanezumab. Median MMDs decreased from 11 (IQR 8–14) at baseline to 4 (IQR 2–5) at Month 3 (*p* < 0.001 vs. baseline) and 3 (IQR 2–6) at Month 6 (*p* < 0.001 vs. baseline). Median MDI decreased from 15 drug intakes (IQR 11–20) at baseline to four drug intakes (IQR 2–7) at Month 3 (*p* < 0.001) and four drug intakes (IQR 2–7) at Month 6 (*p* < 0.001).The corresponding MDIs for MS medications were 10 (IQR 6–14) at baseline, 3 (IQR 1–5, *p* < 0.001) at Month 3, and 2 (IQR 0–4, *p* < 0.001) at Month 6. Monthly drug intakes for non-MS medications were 4 (IQR 0–9) at baseline, 1 (IQR 0–3, *p* < 0.001) at Month 3 and at Month 6.Median MMI decreased from 1.32 (IQR 1.11–1.68) at baseline to 1.00 (IQR 1.00–1.50, *p* < 0.001) at Month 3 and 1.00 (IQR 1.00–1.34, *p* < 0.001) at Month 6.

**Conclusions:**

We confirmed that MoAbs acting on CGRP pathway decrease acute migraine medication consumption. We proposed a new index that can be easily applied in clinical practice to quantify migraine burden and its response to acute medication. Our index could help optimizing migraine acute treatment in clinical practice.

## Introduction

Migraine ranks third as the most prevalent disorder in the world and represents the first cause of disability worldwide in both males and females under the age of 50 (1).

Acute treatments for migraine include migraine-specific (MS) medications, namely triptans and ergots, and non-migraine-specific (non-MS) medications such as non-steroidal anti-inflammatory drugs (NSAIDs), opioids, combined, and simple analgesics. The consumption of both MS medications and non-MS medications is often not appropriate and associated with limited control of migraine episodes (2), together with poor tolerability and adverse events (3, 4). Acute medication overconsumption is one of the most relevant problems in migraineurs as it may favor migraine chronification and lead to the development of medication overuse headache (MOH) (5).

Consumption of acute medication could be an important indicator of the efficacy of migraine preventive treatments. Preventive treatments aim at decreasing the frequency, intensity, duration of attacks and, consequently, the consumption of acute treatments (6). Monoclonal antibodies acting on the calcitonin gene-related peptide (CGRP) pathway (CGRP-MoAbs) represent the first preventive agents specifically designed for migraine (7, 8). The effectiveness of those agents in decreasing the consumption of both MS and non-MS medications has been demonstrated in a subgroup analysis of randomized controlled trials (9). However, detailed real-world data are poorly considered, to date (10).

When assessing acute medication consumption in patients with migraine, there is a difference between the number of drug intakes and the number of days in which the drug is taken. Patients with migraine may take multiple drug intakes of medication in 1 day to treat severe and long-lasting attacks. On the other hand, patients with mild and short-lasting headaches might not take any acute medication on some headache days. Thus, the discrepancy between the drug intakes of drugs taken and the days during which they are taken could be an indirect but simple indication of migraine duration, severity, and response to acute medication.

In the present study, we aimed to report the effectiveness of CGRP-MoAbs on the consumption of MS and non-MS medications in a real-world setting and to present an index of acute medication consumption, the Migraine Medication Index (MMI).

## Methods

### Study Design and Patients

We performed a retrospective observational, multicenter study in two different Czech hospitals, i.e., “Motol University Hospital” and “Military University Hospital,” both in Prague. As a retrospective clinical audit on anonymized clinical practice data, the study was exempt from ethical approval and patients did not have to sign an informed consent.

Inclusion criteria were the following: age ≥18 years old; diagnosis of chronic migraine (CM) or episodic migraine (EM) with or without aura and with or without medication overuse (MO), assessed according to the criteria of the International Classification of Headache Disorders (ICHD-III) (11); >4 monthly migraine days (MMD) and ≥2 previous preventive treatments failed or not tolerated, based on the criteria established by the European Headache Federation (12) and the American Headache Society (13) and Czech regulations for the prescription of monoclonal antibodies acting on the CGRP pathway. With a different approach if compared with public reimbursement established by other countries treatment with CGRP-MoAbs was reimbursed to patients by insurance companies according to the described criteria.

The study population included all patients treated with at least one dose of MoAbs and followed up for at least 6 months; drug discontinuation before 6 months was recorded as well as its reasons (patient decision due to perceived ineffectiveness, adverse events, loss to follow-up).

Patients received subcutaneous administrations of erenumab 140 mg monthly or fremanezumab 225 mg monthly; galcanezumab was not available in the study centers during the inclusion period. All treatments followed common clinical practice; acute treatment withdrawal was not performed in patients with MO. Treatment prescriptions continued despite the outbreak of the SARS-CoV-2 pandemic as the study centers did not close during that period. Following the clinical practice of the study centers, all patients already had a migraine diary, where they reported the number of migraine days, drug intakes, and type of symptomatic drugs assumed to treat migraine; patients continued to record these data throughout the treatment period.

### Data Collection

At baseline visit, for each included patient, we recorded sex, age, comorbidities, history of SARS-CoV-2 infection, family history of migraine, age at migraine onset, migraine duration (years), previous preventive treatment failed or not tolerated (number and type), MMDs in the past 3 months, monthly number of drug intakes and type of symptomatic drugs in the past 3 months, and migraine impact on daily activities, assessed by asking patients to fulfill the Czech version of the Headache Impact Test (HIT-6) (14).

During each monthly follow-up visit, we collected: MMDs in the last month, monthly number of drug intakes and type of symptomatic drugs to treat migraine in the last month, adverse events, and SARS-CoV-2 infection. Moreover, at 3rd and 6th month, we asked patients to fulfill the HIT-6.

### Study Outcomes

Primary endpoints included the decrease in monthly drug intake (MDI), monthly MS medications intake, and monthly non-MS medications intake at each month from baseline. Baseline was defined as a mean of the last 3 months before starting erenumab or fremanezumab treatment. We also computed the MMI by dividing MDI by MMDs; hence, MMI values <1.00 indicate that some migraines are so mild that they do not require acute treatment, while the highest values indicate a high need for acute medication on an average MMD. MMI change at 6 months was computed in patients with <50 and ≥50% decrease in MMDs at 6 months and in those with <50 and ≥50% decrease in MDI at 6 months. Factors potentially influencing MMI change (gender, age, years of migraine history, aura, CM status) were also explored.

Secondary endpoints included the decrease in MMDs from baseline at each month, the proportion of patients who achieved a ≥50% reduction in MMDs from baseline at 3 and 6 months, and the decrease in mean HIT-6 score from baseline at 3 and 6 months of moAb treatment.

### Statistical Analysis

We descriptively synthesized patients' sociodemographic characteristic, comorbidities, migraine diagnosis and history, failed preventive drugs, moAb treatment withdrawal, and adverse events by using absolute numbers and proportions or medians and interquartile ranges (IQRs), as appropriate.

Baseline included the 90-day period preceding treatment with moAbs; monthly follow-ups were performed every 28 days for patients treated with erenumab and every 30 days for those treated with fremanezumab. To ensure comparability, baseline and follow-up variables, including MMDs, MDI, MS, and non-MS medications intake, were all normalized to 30-day periods.

All outcomes were calculated over the total of patients with complete follow-up, irrespective of treatment discontinuation. Patients discontinuing the treatment were considered among those with a <50% reduction in MMDs from baseline. Due to the real-world design of the present study, we could not perform a sample size calculation; all outcomes were exploratory and based on a convenience sample, in the same fashion as previous real-world studies (15–17). We used the Wilcoxon signed-rank test or Spearman's correlation to compare outcome variables.

## Results

### Patients' Characteristics

The whole sample consisted of 90 patients, 27 (30.0%) from the Motol University Hospital and 63 (70.0%) from the Military University Hospital. Most patients were female (82, 91.1%), with a median age of 47 (IQR 42–51) years. Patients' most prevalent comorbidities included cervical spine disorder (23, 25.6%), thyroid disorders (21, 23.3%), cardiovascular disorders (18, 20.0%), and autoimmune disease (17, 18.9%). Of the 90 patients, 75 (83.3%) had episodic and 15 (16.7%) CM. The median age of migraine onset was 16 (IQR 12–24) years, with a median migraine duration of 30 (IQR 21–36) years. Thirty-nine (43.3%) patients had MO (Table 1). Erenumab was administered to 81 (90.0%) patients, while fremanezumab was administered to 9 (10.0%) patients. When patients received the first moAb injection, most of them (66, 73.3%) had ≥3 previous migraine prophylaxis failures. In detail, 84 patients (93.3%) tried unsuccessfully preventive therapy with topiramate, 46 (51.1%) with beta-blockers, 43 (47.8%) with calcium channel blockers, 43 (47.8%) with valproate, 29 (32.2%) with venlafaxine, 26 (28.9%) with amitriptyline (Table 1).

**Table 1:** Characteristics of the study population (*n* = 90).

Female sex, *n* (%)	82 (91.1)
Age, median (IQR)	47 (42–51)
Family history of migraine, *n* (%)	55 (61.1)
Comorbidities, *n* (%)	
Cervical spine disorder	23 (25.6)
Thyroid disorders	21 (23.3)
Cardiovascular disorders	18 (20.0)
Autoimmune diseases	17 (18.9)
Depression	10 (11.1)
Anxiety	8 (8.9)
Type 2 diabetes mellitus	2 (2.2)
Previous SARS-CoV-2 infection, *n* (%)	2 (2.2)
Age at migraine onset, median (IQR)	6 (12-25)
Diagnosis, *n* (%)	
Episodic migraine without aura	59 (65.5)
Episodic migraine with aura	16 (17.8)
Chronic migraine	15 (16.7)
Medication overuse	39 (43.3)
Migraine duration (years), median (IQR)	30 (21-36)
Number of failures, *n* (%)	
2	24 (26.7)
3	35 (38.9)
4	23 (25.5)
>4	8 (8.9)
Preventive treatment failures, *n* (%)	
Topiramate	84 (93.3)
Beta-blockers	46 (51.1)
Calcium channel blockers	43 (47.8)
Valproate	43 (47.8)
Venlafaxine	29 (32.2)
Amitriptyline	26 (28.9)
OnabotolinumtoxinA	8 (8.9)
Pregabalin or Gabapentin	5 (5.6)
Lamotrigine	1 (1.1)
Zonisamide	1 (1.1)

Two patients (2.2%) discontinued the treatment due to perceived treatment ineffectiveness. Discontinuation occurred at 6th month for two patients; no other patient discontinued treatment before. At month 3 of treatment, one patient was prescribed with an add-on therapy (cinnarizine). Only two patients (2.2%) experimented as adverse event local redness or pain at drug site injection.

### Reduction in MMDs and Headache Impact

At baseline, median number of MMDs was 11 (IQR 8–14); it decreased up to 4 (IQR 2–5) and 3 (IQR 2–6) at Month 3 and Month 6, respectively (*p* < 0.001 compared to baseline) (Supplementary Figure 1). At Month 3, 70 patients (77.8%) had a ≥50% reduction in MMDs. At Month 6, the corresponding number was 74 (82.2%). Supplementary Figure 2 shows the details of monthly response rates.

Median HIT-6 score at baseline was 68 (IQR 64–70). The score decreased to 56 (IQR 50–61) at Month 3 (*p* < 0.001 vs. baseline) and 55 (IQR 49–61) at Month 6 (*p* < 0.001 vs. baseline) (Supplementary Figure 3).

### Reduction in MDI, MS, and Non-MS Medications

**Figure 1:**
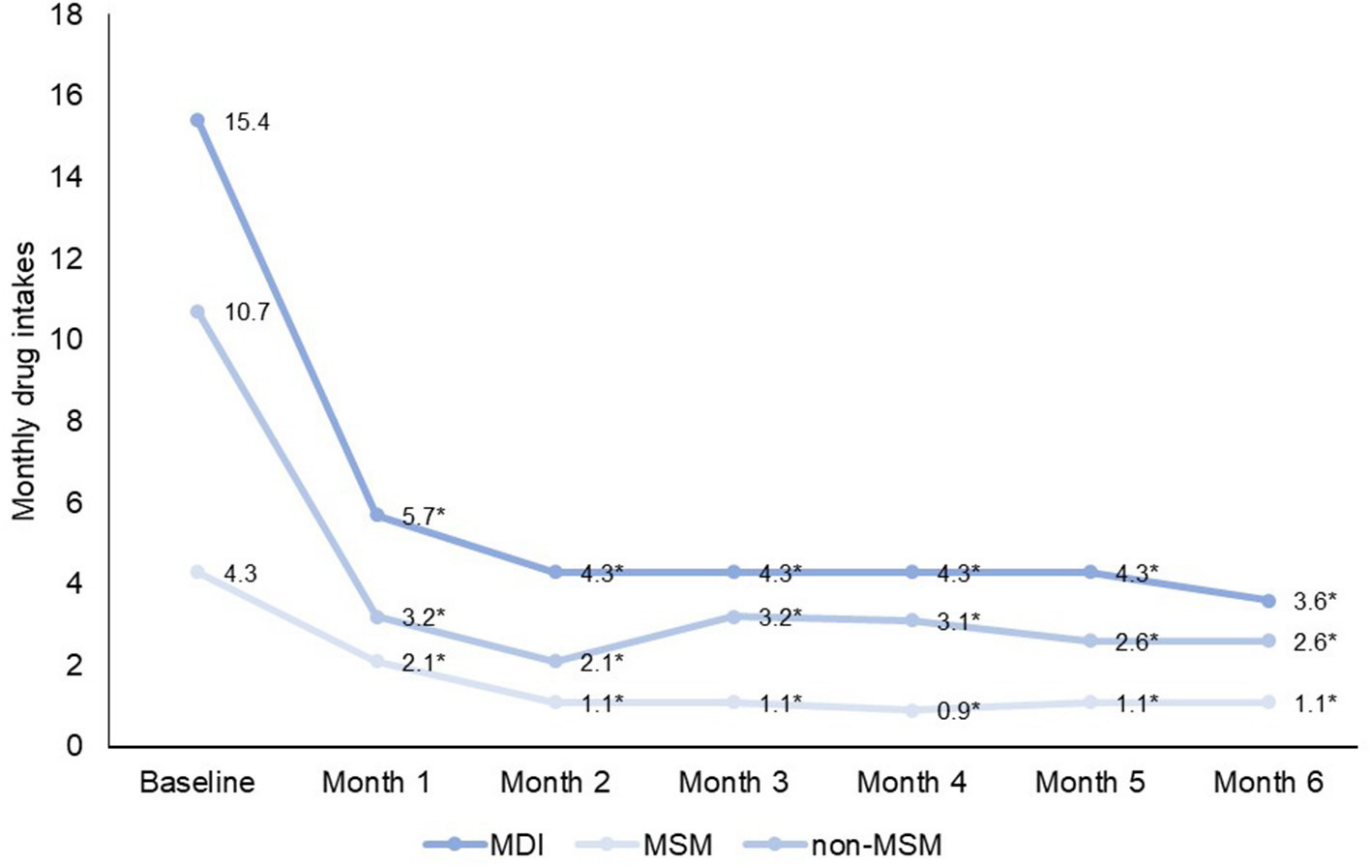
Median Monthly drug intakes (MDI), non-migraine specific medication (NMSM) intakes, and migraine specific medication (MSM) (MS medication) drug intakes. *p ≤ 0.001 compared to baseline for all variables.

Median MDI was 15 (IQR 11–20) drug intakes at baseline and decreased up to 4 (IQR 2–7) drug intakes at both Month 3 and Month 6 (*p* < 0.001 vs. baseline for both comparisons). At Month 3, 73 patients (81.1%) had a ≥50% reduction in MDI. At Month 6, the corresponding number was 79 (87.8%). The median number of MS medication was 10 (IQR 6–14) at baseline; it decreased up to 3 (IQR 1–5) and 2 (IQR 0–4) at Month 3 and Month 6, respectively (*p* < 0.001 vs. baseline for both comparisons). The median number of non-MS medication was 4 (IQR 0–9) at baseline; it decreased up to 1 (IQR 0–3) at both Month 3 and Month 6 (*p* < 0.001 vs. baseline for both comparisons; Figure 1).

### Migraine Medication Index

**Figure 2:**
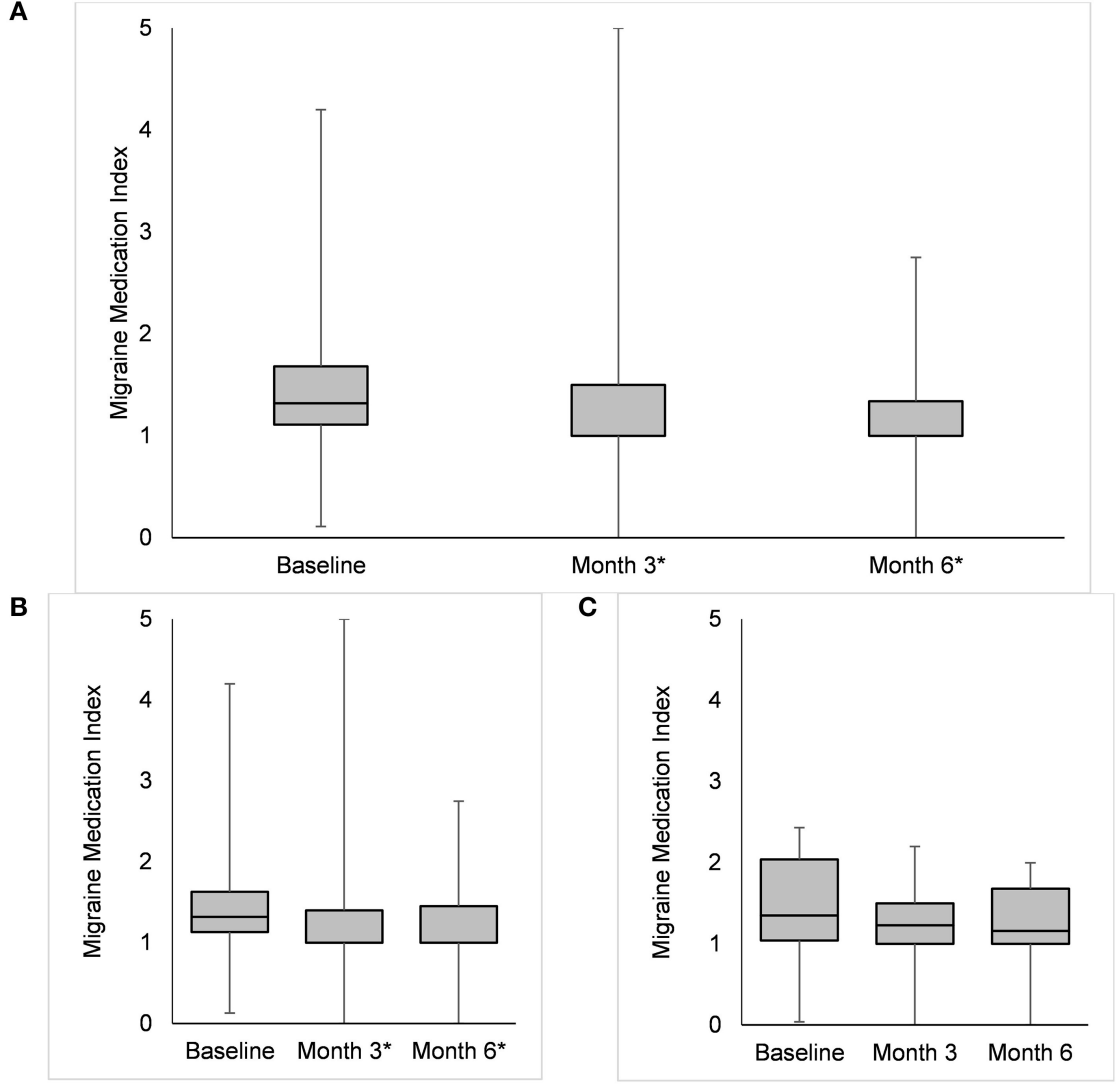
Box plots representing the Migraine Medication Index (i.e., the ratio between monthly drug intake and monthly migraine days) in the overall group **(A)**, in patients reporting a ≥50% reduction in monthly migraine days **(B)**, and in those reporting a <50% reduction in monthly migraine days **(C)** compared with baseline. The asterisks highlight significant (*p* < 0.05) differences.

At baseline, the median MMI was 1.32 (IQR 1.11–1.68); it decreased up to 1.00 (IQR 1.00–1.50; *p* < 0.001) at Month 3 and 1.00 (IQR 1.00–1.34; *p* < 0.001) at Month 6 (Figure 2A). The MMI for MS medications decreased from 1.00 (IQR 0.76–1.68) to 0.76 (IQR 0.00–1.00; *p* < 0.001) at Month 3 and 0.73 (IQR 0.00–1.00; *p* < 0.001) at Month 6 (Figure 2B); the same values for non-MS medications were 0.32 (IQR 0.07–0.76) at baseline, 0.33 (0.00–1.00; *p* = 0.977) at Month 3, and 0.27 (IQR 0.00–1.00; *p* = 0.266) at Month 6 (Figure 2C).

**Figure 3:**
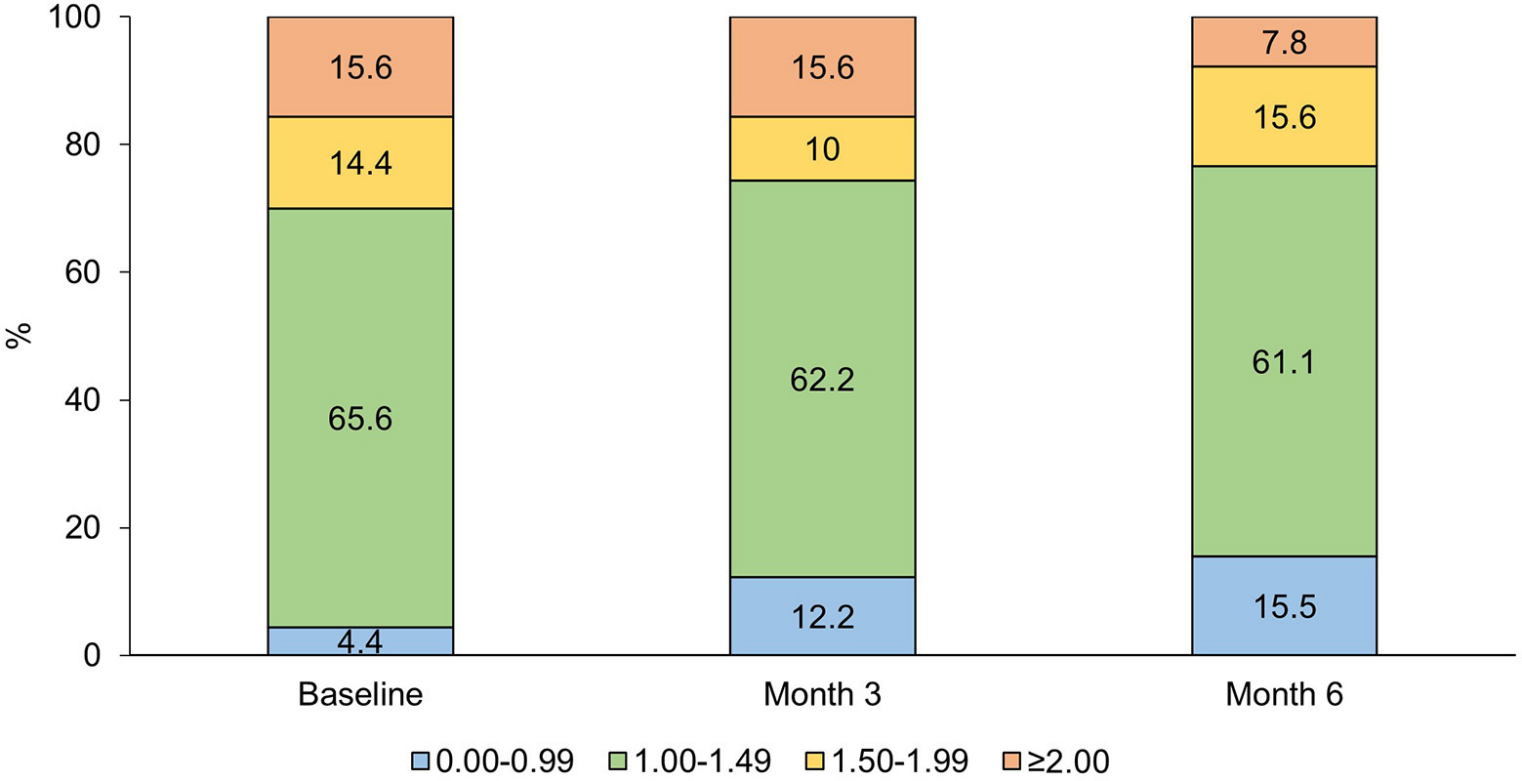
Percent distribution of Migraine Medication Index categories at baseline, Month 3, and Month 6 (*p* < 0.001 for distribution).

We categorized MMI values in four categories: 0.00–0.99, meaning that not all migraines were treated with acute medication; 1.00–1.49; 1.50–1.99; and ≥2.00, meaning that migraine days usually required several acute drug intakes. At baseline, four patients (4.4%) had MMI 0.00–0.99, 59 (65.6%) 1.00–1.49, 13 (14.4%) 1.50–1.99, and 14 (15.6%) ≥2.00; the corresponding numbers were 11 (12.2%), 56 (62.2%), 9 (10.0%), and 14 (15.6%) at Month 3 and 14 (15.5%), 55 (61.1%), 14 (15.6%), and 7 (7.8%) at Month 6 (Figure 3). Although the 0.00–0.99 group increased and the ≥2.00 group decreased numerically over time, the distribution of categories did not change significantly (*P* = 0.144).

The MMI change from baseline to Month 6 did not differ between patients with <50 and ≥50% MMD decrease at Month 6 (−0.27, IQR −0.64 to 0.00, vs. −0.11, IQR −0.74 to +0.18; *P* = 0.390). On the contrary, MMI change was smaller in patients with <50% decrease compared with those with ≥50% decrease in MDI at Month 6 (−0.29, IQR −0.92 to −0.03, vs. +0.03, IQR −0.27 to +0.24; *P* = 0.018).

Migraine Medication Index decrease did not differ according to gender (*P* = 0.160, Wilcoxon test), age (*P* = 0.839, Spearman's correlation), years of migraine history (*P* = 0.326, Spearmen's correlation), aura (*P* = 0.297, Wilcoxon test), or CM status (*P* = 0.770, Wilcoxon test).

## Discussion

One of the main goals of migraine prevention is to limit the use of acute medication (13). Hence, monitoring the use and efficacy of acute medication is an important goal in clinical practice. The decrease in acute medication use was a secondary outcome in most trials of MoAbs acting on the CGRP pathway (18–20) and was the specific object of a subgroup analysis (9). In the present study, we specifically aimed to collect real-life data on the use of MS and non-MS medications and their decrease after treatment with MoAbs acting on the CGRP pathway. However, we also aimed to provide an accurate depiction of the change in acute medication that goes beyond simple dose counting. Effective migraine prevention can indeed decrease the duration and intensity of migraine, thus leading to a further decrease in the drug intakes of acute medication required. For that reason, we deemed useful to consider the number of acute medication intakes together with the number of migraine days in a new index, the MMI. We found that the index decreased independently from the decrease in MMDs, suggesting that acute medication intake decreased in patients treated with erenumab or fremanezumab not only because of a decrease in MMDs, but also because of additional factors. Those factors might include a decrease in the duration and/or intensity of headache, or an increased effectiveness of acute medication; however, this is only a hypothesis, as those variables were not collected in the present study.

Previous studies already considered the importance of outcomes different from the number of MMDs or MDI. A study of patients treated with galcanezumab, a MoAb directed against CGRP, proposed the introduction of total pain burden, which combines migraine frequency, severity, and duration (21). Compared with the total pain burden, the MMI can be easily evaluated as the ratio between two parameters that are commonly assessed in headache diaries, namely MMDs and MDI. Categorizing the MMI can also give an idea of the need of acute medication for each single migraine day. Therefore, this index might provide information not only about the frequency, but also about the efficacy of acute medication. In our opinion, this is an important part of clinical evaluation to adjust patients' medication. Notably, MDI and MMI decreased in different fashions in our study (Figures 1, 2). The proposed category distribution of MMI showed that most patients used one to two acute drug intakes per each MMD, without substantial changes throughout treatment with erenumab or fremanezumab (Figure 3).

We tested the applicability and value of the newly proposed MMI in a real-life study of patients with migraine treated in two centers. The present study mostly included patients with EM, at variance with other available real-life studies on the effectiveness of MoAbs acting on the CGRP pathway, which included only (15, 17, 22, 23) or almost only (16) patients with CM. This difference can be explained by the fact that the present study was performed more recently than the similar ones, at a time when the experience with MoAbs encouraged the spread of MoAb use to patients with high-frequency EM. As regards patients' failed preventive treatments, we considered Czech regulations that were in line with the definition of European Headache Federation and the American Headache Society (i.e., ≥ 2 previous preventive treatments failed or not tolerated) and similar previous trials (13, 18, 19). These criteria are different from those adopted in some countries such as Italy, where MoAbs acting on the CGRP pathway can be prescribed and reimbursed only for patients reporting ≥3 previous preventive treatments failed or not tolerated. However, also in our sample most of the patients (73.3%) had ≥3 previous migraine prophylaxis failures, highlighting the high presence of EM patients with a long treatment history who had not yet found an effective treatment before MoAbs acting on the CGRP pathway.

The strength of our study is that it was conceived to collect complete data about migraine acute medication, including the number of drug intakes of both MS medication and non-MS medication. To our knowledge, this is the first study to provide a complete account of the intake of different medication classes over a 6-month period. However, our data are limited by the collection of MMDs only, while collecting all headache days would have provided more complete data about the patients' pain. Besides, our study included patients treated with more than one drug, i.e., erenumab and fremanezumab; patients treated with fremanezumab represented a small proportion of the sample. The two drugs are taken at different time intervals (28 days for erenumab and 30 days for fremanezumab). To account for this difference, we normalized each monthly period to 30 days; nevertheless, the differences between the two drugs might have introduced heterogeneity.

## Conclusion

We confirmed that CGRP-MoAbs are helpful in reducing the consumption of acute migraine medication. We proposed a new index that can provide an accurate estimate of medication consumption in patients with migraine and possibly new insights on the migraine burden and patterns of acute medication use. The MMI could be useful to optimize acute migraine medication in clinical practice.

## Data Availability Statement

The raw data supporting the conclusions of this article will be made available by the authors, without undue reservation.

## Ethics Statement

Ethical review and approval was not required for the study on human participants in accordance with the local legislation and institutional requirements. Written informed consent for participation was not required for this study in accordance with the national legislation and the institutional requirements.

## Author Contributions

LS, VC, RO, TN, DČ, JŠ, ZM, and SS: conceptualization. LS, VC, and RO: methodology, software, validation, formal analysis, writing—original draft preparation, and visualization. LS, TN, DČ, JŠ, and ZM: investigation. LS, VC, RO, and SS: data curation. TN, DČ, JŠ, ZM, and SS: writing—review and editing. SS: supervision. LS, VC, RO, TN, DČ, JŠ, and ZM: project administration. All authors have read and agreed to the published version of the manuscript.

## Conflict of Interest

VC had a financial relationship (lecturer or member of advisory board) with Novartis and Teva. RO has received sponsorship to attend meetings from Novartis and Teva. SS had a financial relationship (lecturer or member of advisory board) with Abbott, Allergan, Novartis, Teva, and Eli Lilly. The remaining authors declare that the research was conducted in the absence of any commercial or financial relationships that could be construed as a potential conflict of interest.

## Publisher's Note

All claims expressed in this article are solely those of the authors and do not necessarily represent those of their affiliated organizations, or those of the publisher, the editors and the reviewers. Any product that may be evaluated in this article, or claim that may be made by its manufacturer, is not guaranteed or endorsed by the publisher.
