# Supplementary material for: Acute Medication Use in Patients With Migraine Treated With Monoclonal Antibodies Acting on the CGRP Pathway: Results From a Multicenter Study and Proposal of a New Index
Source: Front Neurol. 2022 Feb 28;13:846717. doi: 10.3389/fneur.2022.846717 (PMC8918478; doi:10.3389/fneur.2022.846717)
Supplement: Supplementary file 1 [file Table_1.DOCX]

Supplementary Material


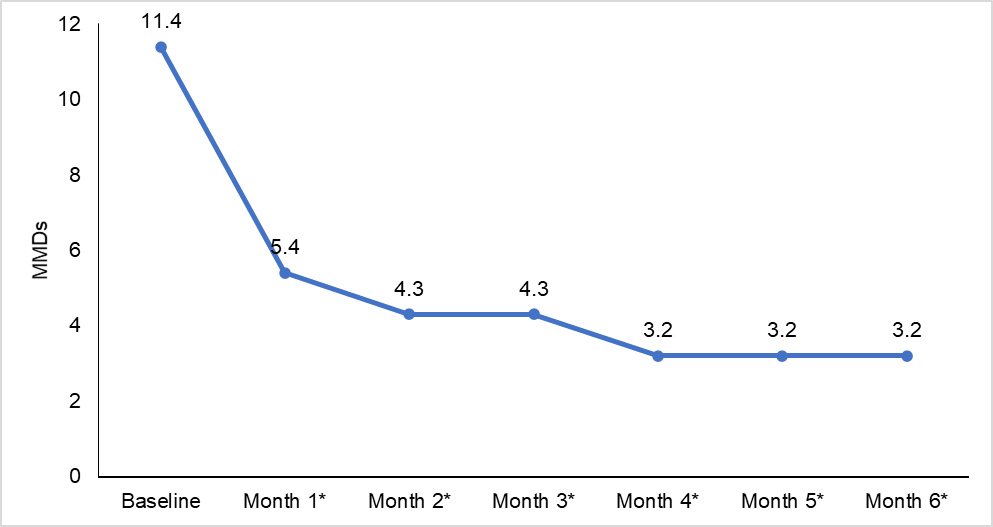


**Supplementary Figure 1.** Decrease in median monthly migraine days (MMDs) in the present study. * p≤0.001 compared to baseline. MMDs indicated monthly migraine days.


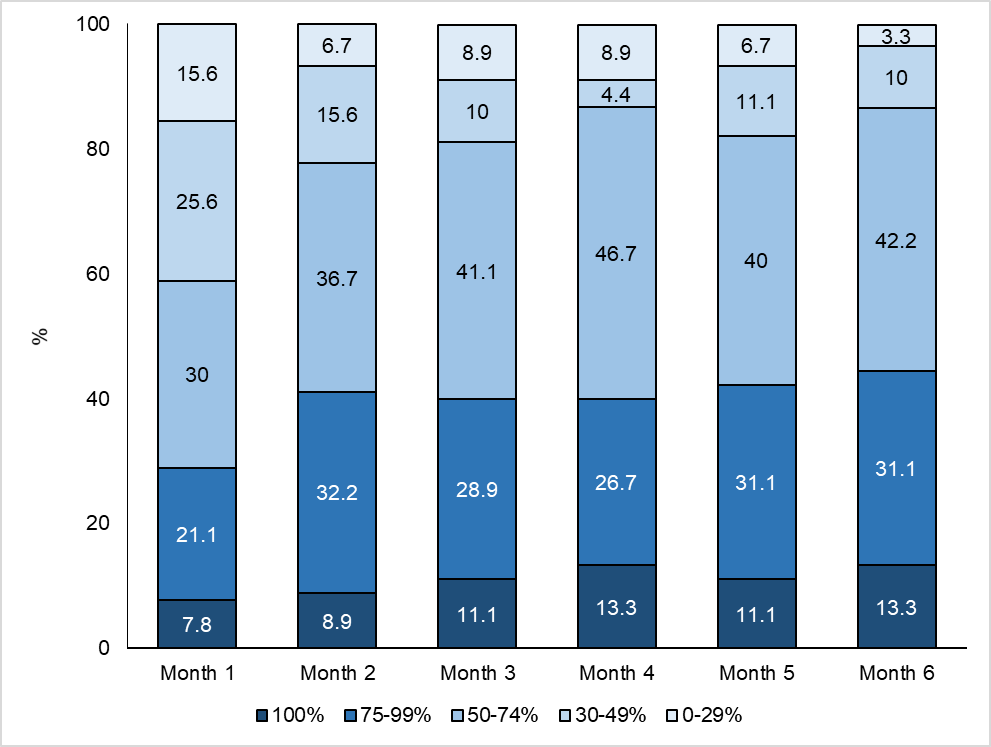


**Supplementary Figure 2.** Percent reduction in monthly migraine days from baseline.


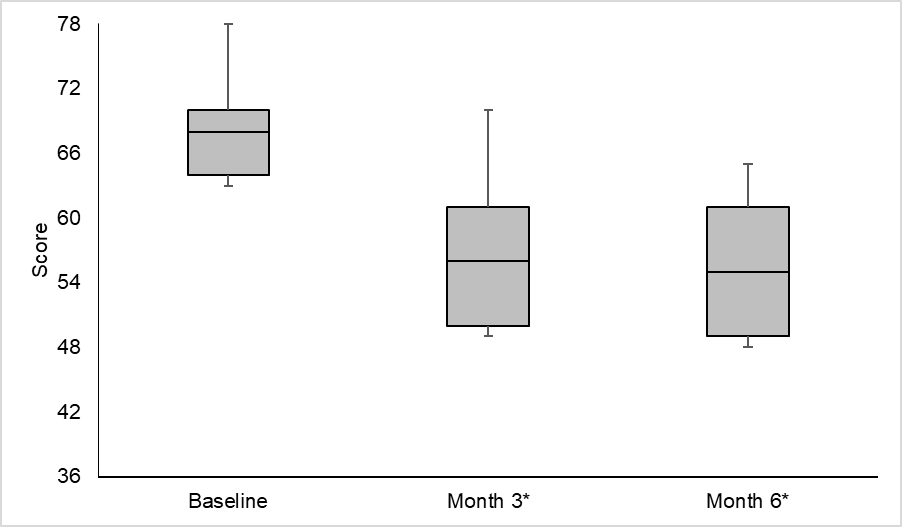


**Supplementary Figure 3.** Reduction in median Headache Impact Test-6 score from baseline to Month 3 and Month 6 in the present study.
